# Abstract choice representations during stable choice-response associations

**DOI:** 10.1038/s42003-025-08129-1

**Published:** 2025-05-14

**Authors:** Katrina R. Quinn, Florian Sandhaeger, Nima Noury, Ema Zezelic, Markus Siegel

**Affiliations:** 1 Department of Neural Dynamics and Magnetoencephalography, Hertie Institute for Clinical Brain Research, University of Tübingen, Tübingen, Germany; 2 Centre for Integrative Neuroscience, University of Tübingen, Tübingen, Germany; 3 MEG Center, University of Tübingen, Tübingen, Germany; 4 Graduate Training Centre of Neuroscience, International Max Planck Research School, University of Tübingen, Tübingen, Germany; 5 German Center for Mental Health (DZPG), Tübingen, Germany

**Keywords:** Perception, Decision

## Abstract

An increasing body of evidence has demonstrated neural representations of choices independent of the motor actions used to report them – so-called abstract choices. However, it remains unclear whether such representations arise due to dynamic changes in choice-response associations or reflect a general property of decision-making. Here, we show that in the human brain, choices are represented abstractly even when choice-response associations remain stable over time. We recorded neural activity using magnetoencephalography while participants performed a motion discrimination task, with choice-response mappings held constant within blocks. We found neural information about participants’ perceptual choices independent of both motor response and visual stimulus. Choice information increased during the stimulus and peaked after the response. Moreover, choice and response information showed distinct cortical distributions, with choice-related signals strongest in frontoparietal regions. Thus, abstract choice representations are not limited to dynamic or action-independent contexts and may be a general feature of decision-making.

## Abstract (web summary)

Human magnetoencephalography reveals neural representations of perceptual choices that are abstracted from motor-responses even during stable choice-response associations. This suggests a general role of abstraction in decision-making.

## Introduction

Many of our every-day decisions are tightly coupled to a particular motor action. Likewise, most of the tasks exploring the neural underpinnings of perceptual decision-making have inextricably linked choices with the motor-responses required to report them^1–5^, for example, in motion discrimination, a rightward eye movement or button press for a downwards motion choice. This is in keeping with one of the dominant accounts of perceptual decision-making, which suggests that choices are embodied^6–8^. This intentional framework suggests that choices emerge as plans to commit a particular action.

However, we are also able to make decisions that cannot immediately be followed by a specific action. This implies that the brain can represent choices independently of actions, which we here refer to as abstract choices. How this is achieved, and under what contexts such representations may persist, has been a matter of continued debate^2,3,6,8–12^. In recent years, there has been a greater focus on uncoupling choices and actions in perceptual tasks, to identify potentially independent neural representations. Indeed, many of these studies have found choice signals which arise before the choice-response mapping is known, therefore abstracting them from any representation of the motor-response^13–17^. More importantly, the same abstract choice signals were identified regardless of whether the choice-response mapping was known in advance or not^17^, indicative of an abstract choice stage across different action contexts.

The extent to which abstract representations of choice occur across different task contexts remains unclear. One possibility is that an abstract choice stage is specifically recruited when flexible shifting between choice-action mappings is required. Previous studies separating choice and action representations have typically used either post-stimulus (action-independent) mapping^14–16,18,19^ or trial-by-trial switching either between action-independent and action-linked decisions^17^ or between different pre-stimulus choice-action mappings^20^. Alternatively, an abstract choice stage may play a general role in decision-making, even when little to no flexibility of choice-action associations is required.

We investigated these alternatives in the human brain by holding the choice-response mapping stable over extended periods of time. Human participants performed an up-down visual motion discrimination task while we recorded neural activity using magnetoencephalography (MEG). Importantly, using a blocked task design and specific analyses, we were able to disentangle choice and response representations. We found neural activity selective for the stimulus, motor response and choice. Crucially, choice information was independent of the choice-response mapping. This abstract choice representation ramped up during the stimulus period, consistent with evidence integration over the motion stimulus, and peaked after the response. Our results show that choices are represented abstractly, even when the association between choice and motor response remains stable over time. This suggests that abstract choice representations may play a more general role than purely action-based frameworks have previously implied.

## Results

### Task and behaviour

**Fig. 1:**
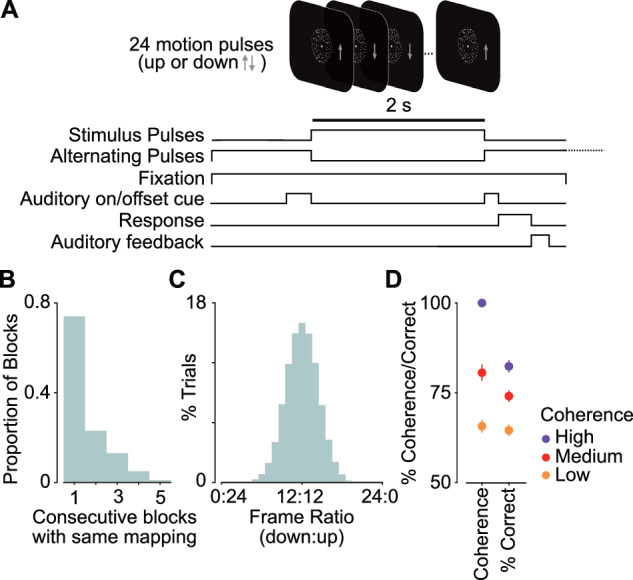
Up-Down motion discrimination task and behavioural performance. **A** On each trial, participants (*n* = 16) were required to discriminate the average motion direction of 24 random-dot motion pulses. Choice was indicated with a left or right button press. The mapping of choice to response was pseudo-randomised block-wise (96 trials per block), such that in one block a left button press corresponded to an ‘up’ choice, and in another block to a ‘down’ choice. Grey arrows depict average pulse motion, shown here for visualisation purposes only. **B** Histogram of the proportion of successive blocks with stable choice-response mapping. **C** Ratio of ‘up’ to ‘down’ motion pulses as a percentage of trials. Each pulse was drawn randomly with equal probability. 12:12 corresponded to an equal number of ‘up’ and ‘down’ pulses on a trial, for which participants were rewarded randomly. **D** Motion coherence and correct performance for the three coherence levels (low, medium, high). Coherence was pseudo-randomly varied across blocks. Low and medium coherence levels were adjusted for each participant to target 66% and 75% correct performance, respectively, using staircases prior to the task. High coherence was 100%. Error bars denote SEM across subjects.

We recorded MEG in 16 human participants (20 prior to exclusions; see Methods) while they performed an up-down visual motion discrimination task (Fig. 1A). On each trial, the participants viewed a stream of 24 random-dot motion pulses (83 ms per pulse). After an auditory go-cue they indicated whether they perceived more up- or downward motion with a left or right button press. The mapping between choice (up or down) and response (left or right button) was manipulated block-wise, such that all trials within a single block of 96 trials had the same choice-response mapping (e.g., left button press for upward motion, right for downward). The mapping was indicated to the participants at the beginning of each block. The mapping for each block was determined pseudo-randomly, such that mappings were balanced across stimulus conditions (see below). Most often, this meant that the choice-response mapping switched between blocks but could also remain stable over multiple blocks (Fig. 1B). We found no significant effect of switching the mapping on overall performance at the beginning of a new block (first 1 trial: *t* = 1.03, *p* = 0.32, cohen’s d = 0.37; first 5 trials: *t* = 1.1, *p* = 0.29, cohen’s d = 0.37; paired t-tests, average proportion correct repeat vs switch blocks).

Each of the 24 motion pulses showed either upward or downward motion. The motion direction was determined randomly on a pulse-by-pulse basis, such that each individual pulse had an equal probability of being an up or downward motion pulse (Fig. 1C). The strength of motion for each pulse was determined by the motion coherence, which was held constant within a block. There were three coherence levels in total – low, medium and high. Low and medium coherence levels were determined by staircases prior to the task to achieve about 66% and 75% performance, respectively. In the high coherence condition, 100% of the dots moved in the specified direction. The order of the coherence blocks was determined pseudo-randomly, such that the coherence levels were balanced.

The coherence staircases had the desired effect that the three coherence levels were well-separated (Fig. 1D, left; mean coherence: 66%, 81%, 100% for low, medium and high coherence, respectively). Behavioural performance corresponded well with the expected performance for the respective coherence levels (Fig. 1D, right; mean correct performance: 65%, 74%, 82% for low, medium and high coherence, respectively).

### Neural information about motion direction and motor responses

We quantified neural information about the direction of motion pulses and about motor responses for each participant using a cross-validated multivariate analysis of variance (cvMANOVA)^17,21,22^. We applied cvMANOVA on preprocessed MEG data across all sensors (see Methods), resulting in a measure of neural information analogous to classifier performance. Importantly, cvMANOVA independently assesses the variability related to a specific variable of interest while excluding confounds related to other variables.

**Fig. 2:**
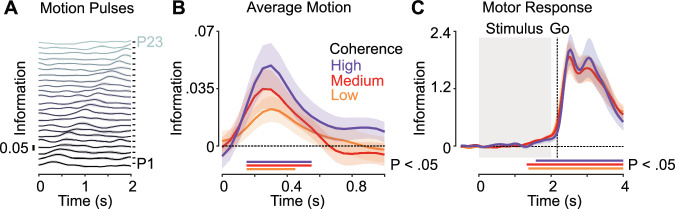
Neural information about motion direction and motor response. **A** Neural information about the direction of each motion pulse averaged across coherence levels. P1 and P23 denote the first and second-to-last pulse respectively. Time axis spans stimulus onset to offset. **B** Neural information averaged across pulses 1-23, aligned to pulse onset, for each coherence level. **C** Neural information about the side on which the button was pressed (left vs. right) for each coherence level. Horizontal bars denote temporal clusters of significant information (*p* < 0.05; cluster permutation, corrected). Solid lines and shaded regions indicate the mean +/− SEM of information across participants (*N* = 16).

We were able to quantify neural information about individual motion pulses, which can be observed as the shifting peaks of information across time relative to stimulus onset (Fig. 2A). When we aligned individual pulses by their onset time and averaged neural information across pulses, we found significant information for each coherence level (Fig. 2B; *p* = 0.03, 0.02, 0.017 for low, medium and high coherence, respectively, cluster permutation statistics). Motion information peaked about 300 ms after pulse onset and continued for several hundred milliseconds. Information about motion direction increased with increasing motion coherence, although this effect was not statistically significant (*F* = 1.23, *p* = 0.30, one-way ANOVA of average pulse information 200–400 ms post pulse onset with factor coherence, eta^2^ = 0.07). As the cvMANOVA also included the subjects’ choice and motor response as factors, neural information about motion pulses was independent of these factors.

We also found significant neural information about motor responses for each coherence level (Fig. 2C; *p* = 0.002, 0.003, <0.001 for low, medium, and high coherence, respectively; cluster permutation). Again, as the cvMANOVA also included the subjects’ choice as a factor, neural information about the response was independent of the choice. Response information started to rise in the second half of the stimulus period, consistent with early motor response preparation, and peaked after the go-cue. We did not observe any differences in the response information as a function of coherence (*F* = 0.01, *p* = 0.99, one-way ANOVA of average response information 0–1 s post stimulus offset with factor coherence, eta^2^ = 0.0007).

In summary, we found robust neural information about the sensory stimulus that subjects had to decide upon and about the motor response with which subjects reported their choice.

### Neural information about choices is independent of responses

We next tested if we could find neural information for the perceptual choice independent of the motor response and stimulus. To do so, we first corrected neural activity for any influence of the stimulus, so that neural activity related to choice would not be confounded by the correlations that arise between stimulus and choice for above-chance performance. We implemented a pulse-based stimulus correction because, unlike for the response or choice variable, merely including the average stimulus motion as a factor in the cvMANOVA would not render other information independent of stimuli at the pulse level (see Methods). Critically, the employed correction was conservative, i.e., would lead to an underestimation of choice information dependent on the behavioural performance. As performance differed between coherence levels, we restricted the subsequent analysis to the data averaged across coherence conditions. Furthermore, as the cvMANOVA also included the response as a factor, choice information was assessed independently from the motor response. In other words, we assessed abstract choice information.

**Fig. 3:**
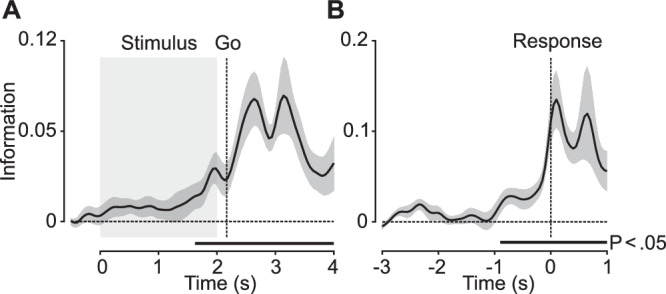
Neural information about choice. **A** Neural information about the participants choices (up vs. down) averaged across coherence conditions and aligned to stimulus onset (*n* = 16). **B** Choice information aligned to the time of response (*n* = 14). Horizontal bars denote temporal clusters of significant information (*p* < 0.05; cluster permutation, corrected). Solid lines and shaded regions indicate the mean +/− SEM of information across participants.

We found significant neural information about the perceptual choice (Fig. 3A; *p* = 0.001, cluster permutation, corrected) which ramped up during the stimulus period (*t* = 2.39, *p* = 0.015 one-tailed *t*-test for the stimulus presentation interval, cohen’s d = 0.58) consistent with evidence integration over the motion pulses and peaked after the go-cue. When we aligned the analysis to the response time based on the reaction time on each trial (Fig. 3B), we found significant choice information (*p* = 0.001, cluster permutation, corrected) that largely peaked right after the response and declined thereafter.

Throughout the experiment, the mapping between choice and response was repeated across some blocks but switched across other blocks. Thus, we tested if abstract choice information differed between blocks with a repeated or switched choice-response mapping. Choice information was significant for both blocks (repeat: *t* = 2.44, *p* = 0.025, cohen’s d = 0.80; switch: *t* = 3.20, *p* = 0.0093, cohen’s d = 1.05; one-tailed t-test 1.5 to 3 s post stimulus onset) and there was no significant difference between blocks (*t* = 0.16, *p* = 0.88, cohen’s d = 0.09; paired t-test 1.5 to 3 s post stimulus onset).

### Choice and response information exhibit distinct cortical distributions

**Fig. 4:**
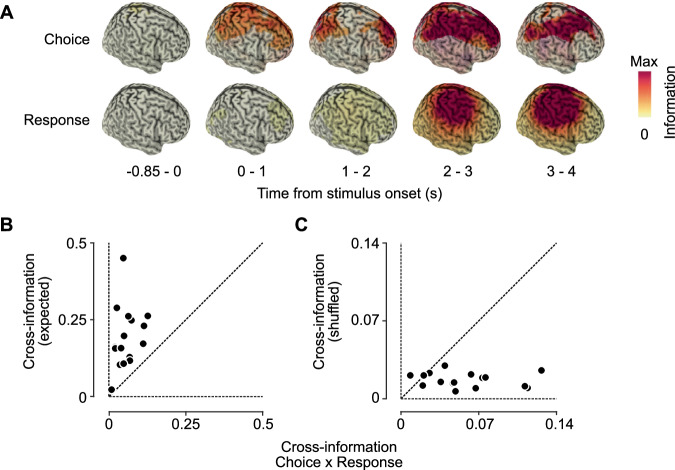
Distinct neural populations underlie choice and response. **A** The spatiotemporal cortical distribution of choice (upper row) and response (bottom row) information for 5 temporal intervals, with respect to stimulus onset (0 s). Only significant clusters of information are shown (*p* < 0.05; cluster permutation). **B** Cross-variable information between choice and response. Absolute cross-variable information was significantly lower than expected for identical neural patterns of choice and response information. **C** Absolute cross-variable information between choice and response was significantly higher than expected by chance for data with shuffled choice labels. Each dot represents a single participant (*n* = 16). All analyses are for the time interval 1.5-3 s post stimulus onset.

To further contrast choice and response information, we employed a searchlight analysis and quantified their cortical distribution across time, averaged across hemispheres (see Methods). We found distinct patterns of information both across space and time (Fig. 4A). For response information, we observed a central peak after the go-cue, which continued well into the response period. In contrast, choice information was strongest in parietal and frontal regions during the stimulus period. This pattern continued into the response period, with additional central contributions.

### Choice information includes response-independent and response-linked components

Our experimental design and analysis approach ensured that the identified choice information was independent of the motor response, i.e., that the identified neural variability that was explained by choices could not be explained by motor responses. However, choice and response information may still recruit overlapping neural populations. To test this, we quantified the cross-variable information between choice and response (see Methods) in an epoch that showed significant information about both variables (1.5 to 3 s post-stimulus onset). We found that the absolute choice-response cross-variable information was significantly lower than expected if they involved perfectly overlapping neural populations (Fig. 4B; z = −3.52, *p* = 0.0004, Wilcoxon signed-rank; Cliff’s δ = -0.84). However, absolute cross-variable information was also significantly above chance, indicating that the representations of choice and response were not completely orthogonal (Fig. 4C; z = 3.21, *p* = 0.0013, Wilcoxon signed-rank; Cliff’s δ = 0.78; real vs. shuffled choice labels). This suggests that choice and response information arise from neural populations that are largely disparate but may have a small degree of overlap.

## Discussion

We found neural information about perceptual choices independent of motor responses in the human brain. Importantly, we did so in a task where the choice-response mappings remained stable over many trials. This contrasts with the previous studies where choice-motor mappings changed on a trial-by-trial basis. In these, choice-motor mappings were either known before stimulus presentations^17,20^, or not^13–19^, but always changed on a trial-by-trial basis. Thus, our results are, to the best of our knowledge, the first to show that abstract choice representations are not limited to tasks that require a quick and flexible mapping of choices onto responses and may therefore reflect a more general property of the perceptual decision-making process.

The fact that we found abstract choice representations even when the choice-response association was stable over time suggests that these representations are more ubiquitous than previously thought. The intentional framework of decision-making has long viewed representations of choice to be synonymous with convergence upon an action plan, framing abstract decision contexts almost as outliers^6,8^. While there has been a wave of evidence for abstract choice representations when the choice-response mapping is not known in advance of the stimulus, only recently has there been evidence to suggest that these persist even when the mapping is known in advance^17,20^. Our findings extend this evidence to a task with a stable choice-response mapping, akin to those commonly used to study action-linked decisions. Thus, our task strongly favoured a purely action-linked decision-making process. Nonetheless, we observed abstract choice information arising from a distinct neural population to that of the response.

Furthermore, our results address a criticism of abstract choice tasks, that participants may still plan response actions even when the mapping is not known in advance^12^. If individuals preferentially associate a specific choice with a specific motor response, this could mean that choice representations that appear to be abstract still reflect motor-linked processes. However, in such a case, the neural populations underlying choice and motor-response would be identical, which is not the case in our data. Whilst we observed some degree of overlap, our cross-variable information analysis shows that even on an individual level, the neural populations supporting choice and motor-response are largely distinct. In addition, if such associations occurred randomly on a trial-by-trial level, the neural patterns supporting both choices would not be separable, and we would therefore not find abstract choice information. Together, these findings contradict a purely intentional framework and suggest that abstract representations of choice are not limited to contexts in which an action cannot immediately be planned.

We found that choice information was present in a network of brain areas distinct from that for the motor response, with parietal and frontal areas showing significant choice signals. These results are broadly consistent with previous findings in non-human primates^13,20^ and humans^14,17,18,23^ that implicate fronto-parietal networks in abstract choice representations. They also complement a recent study, which localised an indirect measure of choice activity to fronto-parietal networks when motor-responses are held stable^24^. Nevertheless, the involvement of specific brain regions in particularly in parietal cortex, remains a topic of ongoing research and debate^11,13,18^.

Our results lead us to several interesting questions. What is the precise role for abstract choice representations when an action can be planned directly? A recent study from Charlton & Goris^20^ found that information about the choice emerged earlier than that about the response, suggestive of a choice stage prior to the action planning. In our study, choice and response information emerged at similar times, although the different strengths of the signals complicates direct timing comparisons. In any case, even without a serial processing of choice and action plan, it could be beneficial to represent choices in an abstract reference frame. For example, when learning to navigate our natural environment, the coupling between particular choices and actions varies with changes of our viewpoint or the dynamic properties of the visual scene. In this case, it could be advantageous to know the perceptual choice associated with particular visual stimuli and outcomes, in addition to the latter’s association with specific actions. Furthermore, evidence from studies on serial dependencies using threshold stimuli has demonstrated an effect of previous perceptual choices on the current choice^25,26^. The fact that the percept itself, independently of the stimulus or the motor-response, can lead to attractive biases suggests that abstract choice representations could contribute to maintain perceptual stability in noisy environments. In addition, since motor-response serial dependencies typically result in repulsive biases^26–28^, there may be distinct mechanisms at work that push and pull to balance perceptual stability with exploration strategies or muscle fatigue.

In which contexts do these abstract choice representations arise? In our study, it was methodologically necessary to change the choice-response mapping several times to measure choice information independently of the visual stimulus and the motor response. While the blocked design was more stable than a trial-by-trial design, it could still be that the infrequent changes of the choice-response mapping led to choices being represented in an abstract format, generalising across block types. This leaves open the possibility that in contexts where the choice and associated action are even more tightly coupled, decision-making operates in a fully intentional way, without abstract choice representations. However, given that our natural behaviour involves many instances of both abstract and action-linked perceptual decisions, it seems intuitive to implement neural processes that allow to fluidly switch between these different contexts. To this end, choices could be represented abstractly across all contexts, potentially simultaneously with action-linked choice signals, and utilised when required.

## Methods

### Participants

20 healthy, right-handed human participants (5 female; mean = 27 years, 4 years SD) took part in the current study and received a monetary reward. All participants had normal or corrected-to-normal vision. Prior to the recording, participants provided written informed consent. The study was approved by the ethical committee of the Medical Faculty and University Hospital of the University of Tübingen and conducted in accordance with the Declaration of Helsinki. All ethical regulations relevant to human research participants were followed.

### Behavioural task and stimuli

Participants performed a symmetric motion discrimination task. On each trial, they were asked to report whether a train of 24 motion pulses contained more upwards or downwards motion. Responses were made with a left- or right-hand button press. Importantly the mapping between choice and motor response varied block-wise, such that a left button press would correspond to an up or down choice depending on the block.

At the start of each trial, participants were required to fixate a small white point at the centre of the screen and continued fixating for the duration of the trial. Once they obtained fixation, 16 alternating up/down motion pulses (1/12 = 0.08333 s pulse duration) were presented (1.33 s total duration), centred around fixation. For the last 0.3333 s of this pre-stimulus pulse train, an auditory onset cue signalled the start of the stimulus pulses. Subsequently, 24 stimulus pulses (1/12 = 0.08333 s pulse duration) were presented (2 s total stimulus duration), each showing more up or downward motion. After the stimulus pulses, a post-stimulus pulse train of alternating up/down pulses began. At the same time, an auditory offset cue (0.1667 s duration) signalled the end of the stimulus-train and acted as a go-cue for participants to respond. After the response, participants received auditory feedback about their performance. Following the feedback, a random even number of alternating up-down pulses (4-14 pulses) was presented before the next trial began.

Participants sat at a viewing distance of 50 cm from the screen. Fixation occurred within a window of 2 degrees (7 subjects) or 1.34 degrees (9 participants) of visual angle. The fixation dot was a white dot with a radius of 0.1 degrees. A single motion pulse consisted of a random-dot kinematogram with 700 white dots, each with a radius of 0.1 degrees. The dots were presented within a circular aperture on a black background (6.7 degrees radius), with an additional circular aperture without dots surrounding the fixation point (2 or 1.34 degrees radius). Each dot moved either upward or downward at 10 degrees per s. The proportion of dots moving upwards vs. downwards was determined by the motion coherence of which there were three levels – low, medium and high. At the high level, all dots moved in one direction. Low and medium coherence levels were determined using two 2:1 staircases (144 trials each) prior to each experiment, to achieve about 66% and 75% correct motion discrimination performance, respectively. For each coherence level, there were 10 possible ‘up’ pulses, which were flipped to produce 10 possible ‘down’ pulses. During the experiment, for each of the 24 stimulus pulses, the pulse direction and specific pulse (1 of 10) were drawn randomly with equal probability. For stimuli with the same number of ‘up’ and ‘down’ pulses (12:12), subjects received randomized feedback.

The auditory onset and offset tones had a frequency of 200 and 300 Hz, respectively. The auditory feedback consisted of two tones: one linearly increasing from 200 to 600 Hz and the other in the reverse direction. The mapping of the two tones to feedback (i.e., correct or incorrect) was varied block-wise.

Each block consisted of 96 trials. Participants typically completed 4 repetitions of each of the 3 types of coherence level blocks, totalling 12 blocks. Each repetition included one block at each coherence level. The choice-motor mapping, auditory feedback mapping, and coherence level for each block were determined pseudo-randomly, and participants were informed of the choice-motor and feedback mappings at the beginning of each block.

### Setup and recording

Neural activity was recorded using a 275-channel whole-head MEG system (Omega 2000, CTF Systems, Port Coquitlam, Canada) at a sampling rate of 2343.75 Hz. Participants sat upright in a dark, magnetically shielded chamber. Stimuli were projected onto a screen using either an LCD projector (Sanyo PLC-XP41, Moriguchi, Japan) or a DLP LED PROPixx projector (VPixx, Saint-Bruno, Canada) at 60 Hz refresh rate. Eye movements were tracked with an eyetracking system (Eyelink 1000, SR Research) at a sampling rate of 1000 Hz.

### Preprocessing

MEG Data was low-pass filtered at 10 Hz (two-pass forward-reverse Butterworth filter, order 4) and down-sampled to 20 Hz. We used robust detrending^29^ to remove polynomial trends from the MEG data in a piece-wise fashion (600 s pieces, removal of linear trend followed by 10th order polynomial), and baseline-corrected each trial (−0.83 s to −0.33 s). Individual noisy channels and trials were defined as those exceeding 10 times the standard deviation of the variability across channels or trials, respectively, and excluded from the analysis. For all temporally resolved analyses, results were smoothed using a 100 ms Hanning window (full width at half maximum).

### Data exclusion

Trials with blinks or eye movements outside of the fixation window were aborted and thus automatically excluded from analysis. Eye movements could not be measured for three participants, but their exclusion did not significantly change the results.

For some participants, we were unable to analyse all 12 blocks due to technical issues. In one case, this led to the exclusion of an entire coherence condition (high/full) due to a lack of counterbalancing across choice-response mappings.

Two participants were excluded due to insufficient behavioural performance caused by a misunderstanding of the task. Two participants were excluded due to technical problems with stimulus presentation or data recording. For reaction-time aligned choice analysis (Fig. 3B), two participants were excluded due to technical issues.

### Source reconstruction

As MRIs for individual participants were not available, we sourced and reconstructed the data using a standard MNI template brain. We generated a single-shell head model^30^ and for each participant estimated three-dimensional (x, y, and z-direction) MEG source activity at 457 equally spaced locations 7 mm beneath the skull, using linear spatial filtering (beamforming)^31^. We retained, for each source, activity in all 3 directions. For the searchlight analysis, we used each of the 457 sources’ immediate neighbours, including all 3 dipole directions. We averaged data within 5 intervals with respect to stimulus onset (−0.85–0 s; 0–1 s; 1–2 s; 2–3 s; 3–4 s).

### Cross-validated MANOVA

We applied a cross-validated MANOVA (cvMANOVA) on the MEG data from single participants to estimate neural information about each of the variables of interest^21,32^. cvMANOVA constitutes an extension of the commonly used cross-validated Mahalanobis distance and allows for the simultaneous estimation of neural data variability due to several variables of interest. This estimation is performed in relation to unexplained noise variability. We therefore first estimate a baseline noise covariance matrix, using all trials from all possible unique combinations of variables or ‘conditions’. For each unique condition, beta weights are estimated and contrasted between conditions in cross-validation fold ‘training’ and ‘test’ sets separately. An estimate of true pattern distinctness is computed as the dot product of these contrasts, normalised by the noise covariance:

$$D={trace}\left(\frac{1}{n}{{B}^{{\prime} }}_{{train}}{C}_{{train}}{C}_{{train}}^{-1}{{X}^{{\prime} }}_{{test}}{X}_{{test}}{C}_{{train}}{C}_{{test}}^{-1}{B}_{{test}}{\varSigma }^{-1}\right)$$

where X_test_ is the design matrix indicating the unique condition of each trial in the test set, C_train is_ the contrast vector the model is trained on, C_test_ the test contrast vector and Σ^-1^ the inverted noise covariance matrix. B_train_ and B_test_ contain the regression parameters of a multivariate general linear model:

$${B}_{{train}}={X}_{{train}}^{-1}{Y}_{{train}}$$

$${B}_{{test}}={X}_{{test}}^{-1}{Y}_{{test}}$$

where Y_train_ and Y_test_ are the training and test datasets. The inverted noise covariance matrix was estimated from the mean activity during the time period starting from −0.85 seconds pre-stimulus to 2 seconds post-stimulus:

$${B}_{{{train}}_{{avg}}}={X}_{{train}}^{-1}{Y}_{{{train}}_{{avg}}}$$

$$\Xi = {Y}_{{{train}}_{{avg}}}{X}_{{train}}{B}_{{{train}}_{{avg}}}$$

$${\Sigma }^{-1}=\left({fE}-p-1\right)\cdot {({\Xi }^{\prime} {\Xi })}^{-1}$$

With fE being the degrees of freedom and p the number of sources used. Ξ was regularised towards the unity matrix using a regularisation parameter of 0.05.

Given that the design matrix and contrast vector include all unique conditions i.e., all possible combinations of variable levels, cvMANOVA independently quantifies information about each variable of interest, while not being confounded by information about the other, potentially correlated variables. In other words, cvMANOVA quantifies the pattern distinctness explained by each variable after discounting the patterns explained by all other variables included in the model. Importantly, cvMANOVA effectively controls imbalances in the distribution of trials over conditions without explicit stratification and the resulting loss of data.

Prior to cvMANOVA we reduced the dimensionality of the data using PCA. We computed a de-mixing matrix on the condition means of the training data only, and subsequently applied it to the test data. We selected the first 100 components for further analysis.

For all analyses we performed two-fold cross validation and 10 repetitions of cvMANOVA with different random seeds. We averaged results across repetitions and folds.

### Task variables and stimulus correction

To accurately estimate neural information about variables independently of each other, we had to ensure that all combinations of the variables of interest were present, including those under experimental control (coherence, choice-response mapping) and those dependent on the participants behaviour (choice, response). In all cvMANOVAs we included choice, response, and coherence as variables.

As each stimulus pulse was randomly selected from 10 possible movies for each direction, there were many unique 2 s stimuli, such that the full stimulus could not be included as a variable. Given that stimulus and choice are correlated for non-chance performance, we used a stimulus correction procedure for those analyses assessing neural choice information independent of the stimulus (Figs. 3 and 4). For this, we computed the average neural activity for each pulse position, motion direction, and coherence, and subtracted the 24 pulse-related averages from the neural data of individual trials based on the pulses presented on these trials. We then added back the pulse-related activity averaged across motion directions, independently for pulse position and coherence. This conservative correction likely removes choice-related neural activity that is strongly correlated with the stimulus. As this results in different over-corrections depending on performance, we did not interpret differences in choice information between coherence conditions.

To estimate the neural information associated with each individual pulse, we computed cvMANOVAs with the additional variable pulse direction, for each pulse position independently.

### Cross-variable information

To assess whether choice and response shared a common representational space, we measured cross-variable information. We implemented this by using a training contrast C_train_ differentiating between choice levels, and a test contrast C_test_ differentiating between response levels, and vice versa.

Notably, a possible relationship between choice and response representations could vary in directionality across participants, such as for example arbitrary choice-response associations that is unrelated to the actual choice-response mapping (e.g. an association between the left button and upwards motion). Thus, we did not average the resulting cross-variable information metric but compared absolute cross-variable information across participants (Fig. 4B, C).

Given that the maximal amount of shared information between two variables depends on the information available for each variable independently, it was important to take the strength of the individual representations into account. We therefore compared the measured cross-variable information to an estimate of the cross-variable information that could be expected for identical representations of variable strength^32^

$${E}_{{ChRs}}=\,\sqrt{\left|{ChRs}\right|}\cdot {sign}\left({Ch}\right)\cdot {sign}({Rs})$$

where Ch and Rs denote the pattern distinctness for choice and response, respectively, and E_ChRs_ is the expected cross-variable information. If representations were identical, the measured cross-variable information is expected to approach E_ChRs_, whereas cross-variable information smaller than E_ChRs_ indicates non-overlapping representations^32^.

### Statistics and reproducibility

We recorded 20 participants in-keeping with the range commonly used in MEG studies. Due to data exclusions, statistical analyses were performed over 16 participants, except for the response-aligned choice information (*n* = 14).

We assessed the statistical significance of information using cluster-based permutation tests. After determining temporally contiguous clusters during which pattern distinctness was higher than 0 (one-tailed t-test over participants, *p* < 0.05), we randomly multiplied the information time-course of each participant 1000 times with either 1 or −1. In each random permutation, we recomputed information clusters and determined the maximum cluster mass. Each original cluster was assigned a p-value by comparing its mass to the distribution of the random permutation’s maximum cluster masses. Importantly, the comparison against the maximum cluster mass distribution corrects for multiple comparisons across time^33^.

To assess the cortical distribution of neural information, we performed analogous cluster permutation tests, but across space and time.

To test for differences between information as a function of coherence condition, we used one-way ANOVAs. We did this for average pulse information in the time 200–400 ms post-pulse onset, and for response information 0–1 s post-stimulus offset. We additionally used a one-tailed t-test to test for significant choice information specifically during the stimulus period (0–2 s post stimulus onset). For ANOVAs and the t-test we computed effect sizes using eta^2^ and Cohen’s D, respectively.

To determine whether the multivariate patterns underlying choice and response were significantly different, we used Wilcoxon signed-rank tests, after determining that the absolute cross-variable information was not normally distributed (according to the Kolmogorov-Smirnov test). We tested whether the absolute cross-variable information was smaller than the expected cross-variable information, and whether the absolute cross-variable information was higher than would be expected by chance. For the latter, we ran cvMANOVA on data with shuffled choice labels and tested the absolute cross-variable information against this value. Tests were performed on the time period with robust choice and response information across participants (1.5–3 s). For Wilcoxon-signed rank tests we computed effect sizes using Cliff’s δ.

To test if choice information differed between blocks with repeated or switched choice-response mapping cvMANOVA was separately performed for these blocks within a limited subset of the data (7 subjects with 1 coherence condition each). This subset resulted from the requirement of at least two ‘repeat’ and two ‘switch’ blocks (one of each choice-response mapping) within a coherence condition per participant, which was only the case for 7 out of 16 participants. Furthermore, within these 7 participants, the balancing of blocks resulted in only being able to use data from single coherence conditions.

### Software

Experimental code was written in MATLAB (Mathworks) using custom code and Psychophysics Toolbox extensions^34^. All analyses were performed in MATLAB using custom code as well as the Fieldtrip toolboxes^35^.

### Reporting summary

Further information on research design is available in the Nature Portfolio Reporting Summary linked to this article.

## Supplementary information

Reporting Summary

## Data Availability

Preprocessed MEG data is available from the authors upon reasonable request.

## Code availability

Analysis code to reproduce all reported results is available from the authors upon reasonable request.

## References

[1] Donner, T. H., Siegel, M., Fries, P. & Engel, A. K. Buildup of choice-predictive activity in human motor cortex during perceptual decision making. *Curr. Biol.* **19**, 1581–1585 (2009). 19747828 10.1016/j.cub.2009.07.066

[2] Gold, J. I. & Shadlen, M. N. The neural basis of decision making. *Annu. Rev. Neurosci.* **30**, 535–574 (2007). 17600525 10.1146/annurev.neuro.29.051605.113038

[3] Heekeren, H. R., Marrett, S. & Ungerleider, L. G. The neural systems that mediate human perceptual decision making. *Nat. Rev. Neurosci.* **9**, 467–479 (2008). 18464792 10.1038/nrn2374

[4] Shadlen, M. N. & Newsome, W. T. Neural basis of a perceptual decision in the Parietal Cortex (Area LIP) of the Rhesus Monkey. *J. Neurophysiol.* **86**, 1916–1936 (2001). 11600651 10.1152/jn.2001.86.4.1916

[5] Siegel, M., Buschman, T. J. & Miller, E. K. Cortical information flow during flexible sensorimotor decisions. *Science* **348**, 1352–1355 (2015). 26089513 10.1126/science.aab0551 PMC4721574

[6] Cisek, P. & Kalaska, J. F. Neural mechanisms for interacting with a world full of action choices. *Annu. Rev. Neurosci.* **33**, 269–298 (2010). 20345247 10.1146/annurev.neuro.051508.135409

[7] O’Regan, J. K. & Noë, A. A sensorimotor account of vision and visual consciousness. *Behav. Brain Sci.* **24**, 939–973 (2001). 12239892 10.1017/s0140525x01000115

[8] Shadlen, M. N., Kiani, R., Hanks, T. D. & Churchland, A. K. Neurobiology of Decision Making: An Intentional Framework. in *Better Than Conscious?* (eds. Engel, C. & Singer, W.) *MIT Press*, 71–102 (2008).

[9] Cisek, P. Making decisions through a distributed consensus. *Curr. Opin. Neurobiol.* **22**, 927–936 (2012). 22683275 10.1016/j.conb.2012.05.007

[10] Wispinski, N. J., Gallivan, J. P. & Chapman, C. S. Models, movements, and minds: bridging the gap between decision making and action. *Ann. N. Y. Acad. Sci.* **1464**, 30–51 (2020). 30312476 10.1111/nyas.13973

[11] Shushruth, S., Zylberberg, A. & Shadlen, M. N. Sequential sampling from memory underlies action selection during abstract decision-making. *Curr. Biol.* **32**, 1949–1960 (2022). 35354066 10.1016/j.cub.2022.03.014 PMC9090972

[12] Okazawa, G. & Kiani, R. Neural mechanisms that make perceptual decisions flexible. *Annu. Rev. Physiol.* **85**, 191–215 (2023). 36343603 10.1146/annurev-physiol-031722-024731 PMC10308708

[13] Bennur, S. & Gold, J. I. Distinct representations of a perceptual decision and the associated oculomotor plan in the monkey lateral intraparietal area. *J. Neurosci.* **31**, 913–921 (2011). 21248116 10.1523/JNEUROSCI.4417-10.2011 PMC3380543

[14] Hebart, M. N., Donner, T. H. & Haynes, J.-D. Human visual and parietal cortex encode visual choices independent of motor plans. *NeuroImage* **63**, 1393–1403 (2012). 22922368 10.1016/j.neuroimage.2012.08.027

[15] Horwitz, G. D., Batista, A. P. & Newsome, W. T. Representation of an abstract perceptual decision in Macaque superior colliculus. *J. Neurophysiol.* **91**, 2281–2296 (2004). 14711971 10.1152/jn.00872.2003

[16] Quinn, K. R., Seillier, L., Butts, D. A. & Nienborg, H. Decision-related feedback in visual cortex lacks spatial selectivity. *Nat. Commun.* **12**, 4473 (2021). 34294703 10.1038/s41467-021-24629-0 PMC8298450

[17] Sandhaeger, F., Omejc, N., Pape, A.-A. & Siegel, M. Abstract perceptual choice signals during action-linked decisions in the human brain. *PLoS Biol.* **21**, e3002324 (2023). 37816222 10.1371/journal.pbio.3002324 PMC10564462

[18] Filimon, F., Philiastides, M. G., Nelson, J. D., Kloosterman, N. A. & Heekeren, H. R. How embodied is perceptual decision making? Evidence for separate processing of perceptual and motor decisions. *J. Neurosci.* **33**, 2121–2136 (2013). 23365248 10.1523/JNEUROSCI.2334-12.2013 PMC6619122

[19] Ludwig, S., Herding, J. & Blankenburg, F. Oscillatory EEG signatures of postponed somatosensory decisions. *Hum. Brain Mapp.* **39**, 3611–3624 (2018). 29717524 10.1002/hbm.24198 PMC6866617

[20] Charlton, J. A. & Goris, R. L. T. Abstract deliberation by visuomotor neurons in prefrontal cortex. *Nat. Neurosci.* **27**, 1167–1175 (2024). 38684894 10.1038/s41593-024-01635-1 PMC11156582

[21] Allefeld, C. & Haynes, J.-D. Searchlight-based multi-voxel pattern analysis of fMRI by cross-validated MANOVA. *NeuroImage* **89**, 345–357 (2014). 24296330 10.1016/j.neuroimage.2013.11.043

[22] Voigtlaender, V. A., Sandhaeger, F., Hawellek, D. J., Hage, S. R. & Siegel, M. Neural representations of the content and production of human vocalization. *Proc. Natl Acad. Sci. USA.* **120**, e2219310120 (2023). 37253014 10.1073/pnas.2219310120 PMC10265962

[23] Hebart, M. N., Schriever, Y., Donner, T. H. & Haynes, J.-D. The relationship between perceptual decision variables and confidence in the human brain. *Cereb. Cortex* **26**, 118–130 (2016). 25112281 10.1093/cercor/bhu181

[24] Gherman, S. et al. Intracranial electroencephalography reveals effector-independent evidence accumulation dynamics in multiple human brain regions. *Nat. Hum. Behav.* **8**, 758–770 (2024). 38366105 10.1038/s41562-024-01824-9 PMC12188985

[25] Braun, A., Urai, A. E. & Donner, T. H. Adaptive History Biases Result from Confidence-Weighted Accumulation of past Choices. *J. Neurosci.* **38**, 2418–2429 (2018). 29371318 10.1523/JNEUROSCI.2189-17.2017 PMC5858589

[26] Zhang, H. & Alais, D. Individual difference in serial dependence results from opposite influences of perceptual choices and motor responses. *J. Vis.* **20**, 2 (2020). 32744618 10.1167/jov.20.8.2 PMC7438638

[27] Pape, A.-A. & Siegel, M. Motor cortex activity predicts response alternation during sensorimotor decisions. *Nat. Commun.* **7**, 13098 (2016). 27713396 10.1038/ncomms13098 PMC5059771

[28] Pape, A.-A., Noury, N. & Siegel, M. Motor actions influence subsequent sensorimotor decisions. *Sci. Rep.* **7**, 15913 (2017). 29162928 10.1038/s41598-017-16299-0 PMC5698410

[29] De Cheveigné, A. & Arzounian, D. Robust detrending, rereferencing, outlier detection, and inpainting for multichannel data. *NeuroImage* **172**, 903–912 (2018). 29448077 10.1016/j.neuroimage.2018.01.035 PMC5915520

[30] Nolte, G. The magnetic lead field theorem in the quasi-static approximation and its use for magnetoencephalography forward calculation in realistic volume conductors. *Phys. Med. Biol.* **48**, 3637–3652 (2003). 14680264 10.1088/0031-9155/48/22/002

[31] Van Veen, B. D., Van Drongelen, W., Yuchtman, M. & Suzuki, A. Localization of brain electrical activity via linearly constrained minimum variance spatial filtering. *IEEE Trans. Biomed. Eng.* **44**, 867–880 (1997). 9282479 10.1109/10.623056

[32] Sandhaeger, F. & Siegel, M. Testing the generalization of neural representations. *NeuroImage* **278**, 120258 (2023). 37429371 10.1016/j.neuroimage.2023.120258 PMC10443234

[33] Maris, E. & Oostenveld, R. Nonparametric statistical testing of EEG- and MEG-data. *J. Neurosci. Methods* **164**, 177–190 (2007). 17517438 10.1016/j.jneumeth.2007.03.024

[34] Kleiner, M., Brainard, D. H. & Pelli, D. G. What’s new in Psychtoolbox-3?. *Perception* **36**, 89 (2007).

[35] Oostenveld, R., Fries, P., Maris, E. & Schoffelen, J.-M. FieldTrip: Open Source Software for Advanced Analysis of MEG, EEG, and Invasive Electrophysiological Data. *Comput. Intell. Neurosci.* **2011**, 1–9 (2011). 21253357 10.1155/2011/156869 PMC3021840

