# Supplementary material for: Abstract choice representations during stable choice-response associations
Source: Commun Biol. 2025 May 14;8:752. doi: 10.1038/s42003-025-08129-1 (PMC12078719; doi:10.1038/s42003-025-08129-1)
Supplement: Supplementary file 1 — Reporting Summary [file 42003_2025_8129_MOESM1_ESM.pdf]

nature portfolio

Corresponding author(s): Katrina Rose Quinn
Markus Siegel

Last updated by author(s): Apr 21, 2025

Reporting Summary

Nature Portfolio wishes to improve the reproducibility of the work that we publish. This form provides structure for consistency and transparency in reporting. For further information on Nature Portfolio policies, see our Editorial Policies and the Editorial Policy Checklist.

Statistics

For all statistical analyses, confirm that the following items are present in the figure legend, table legend, main text, or Methods section.

n/a	Confirmed	
☐	☒	The exact sample size (*n*) for each experimental group/condition, given as a discrete number and unit of measurement
☐	☒	A statement on whether measurements were taken from distinct samples or whether the same sample was measured repeatedly
☐	☒	The statistical test(s) used AND whether they are one- or two-sided<br>*Only common tests should be described solely by name; describe more complex techniques in the Methods section.*
☐	☒	A description of all covariates tested
☐	☒	A description of any assumptions or corrections, such as tests of normality and adjustment for multiple comparisons
☐	☒	A full description of the statistical parameters including central tendency (e.g. means) or other basic estimates (e.g. regression coefficient) AND variation (e.g. standard deviation) or associated estimates of uncertainty (e.g. confidence intervals)
☐	☒	For null hypothesis testing, the test statistic (e.g. *F*, *t*, *r*) with confidence intervals, effect sizes, degrees of freedom and *P* value noted<br>*Give P values as exact values whenever suitable.*
☒	☐	For Bayesian analysis, information on the choice of priors and Markov chain Monte Carlo settings
☒	☐	For hierarchical and complex designs, identification of the appropriate level for tests and full reporting of outcomes
☐	☒	Estimates of effect sizes (e.g. Cohen's *d*, Pearson's *r*), indicating how they were calculated

Our web collection on statistics for biologists contains articles on many of the points above.

Software and code

Policy information about availability of computer code

Data collection	MEG data were collected using acquistion software developed by the system manufacturer (CTF Systems Inc.). The experimental task was implemented using Psychtoolbox 3 and MATLAB 2016b.
Data analysis	All data analyses were carried out using MATLAB 2020a, the FieldTrip library and custom code.

For manuscripts utilizing custom algorithms or software that are central to the research but not yet described in published literature, software must be made available to editors and reviewers. We strongly encourage code deposition in a community repository (e.g. GitHub). See the Nature Portfolio guidelines for submitting code & software for further information.

Data

Policy information about availability of data

All manuscripts must include a data availability statement. This statement should provide the following information, where applicable:

- Accession codes, unique identifiers, or web links for publicly available datasets
- A description of any restrictions on data availability
- For clinical datasets or third party data, please ensure that the statement adheres to our policy

Preprocessed MEG data and analysis code to reproduce all reported results is available from the authors upon reasonable request.

Research involving human participants, their data, or biological material

Policy information about studies with human participants or human data. See also policy information about sex, gender (identity/presentation), and sexual orientation and race, ethnicity and racism.

Reporting on sex and gender	From our human volunteers, 5 were of female sex, and 15 of male sex.
Reporting on race, ethnicity, or other socially relevant groupings	We did not systematically control for the race or ethnicity of our participants.
Population characteristics	20 healthy, right-handed human volunteers (5 female; mean=27 years, 4 year SD) participated in this study.
Recruitment	Participants were recruited via mailing lists at the University of Tübingen, as well as from the local community. Some participants were familiar with neuroscience or psychologiy in general, but not with the specific hypotheses of the study.
Ethics oversight	The study was approved by the local ethics committee of the Medical Faculty of the University of Tübingen.

Note that full information on the approval of the study protocol must also be provided in the manuscript.

Field-specific reporting

Please select the one below that is the best fit for your research. If you are not sure, read the appropriate sections before making your selection.

☒ Life sciences ☐ Behavioural & social sciences ☐ Ecological, evolutionary & environmental sciences

For a reference copy of the document with all sections, see nature.com/documents/nr-reporting-summary-flat.pdf

Life sciences study design

All studies must disclose on these points even when the disclosure is negative.

Sample size	No sample size calculation was performed. We used a standard sample size of 20 participants.
Data exclusions	In total, four participants were excluded from all analyses. Two of these were due to insufficient behavioural performance caused by a misunderstanding of the task. The other two were excluded due to technical problems with stimulus presentation or data recording. For reaction-time aligned choice analysis, two further participants were excluded due to technical issues.
Replication	We did not attempt to replicate our findings as the main result was highly statistically robust.
Randomization	We did not use any randomisation as there were no experimental groups.
Blinding	Blinding was not relevant, as there were no experimental groups.

Reporting for specific materials, systems and methods

We require information from authors about some types of materials, experimental systems and methods used in many studies. Here, indicate whether each material, system or method listed is relevant to your study. If you are not sure if a list item applies to your research, read the appropriate section before selecting a response.

Materials & experimental systems

n/a	Involved in the study	
☒	☐	Antibodies
☒	☐	Eukaryotic cell lines
☒	☐	Palaeontology and archaeology
☒	☐	Animals and other organisms
☒	☐	Clinical data
☒	☐	Dual use research of concern
☒	☐	Plants

Methods

n/a	Involved in the study	
☒	☐	ChIP-seq
☒	☐	Flow cytometry
☒	☐	MRI-based neuroimaging

Plants

Seed stocks	NA
Novel plant genotypes	NA
Authentication	NA
